# Identification, Evolution, and Expression Profiling of Histone Lysine Methylation Moderators in *Brassica rapa*

**DOI:** 10.3390/plants8120526

**Published:** 2019-11-20

**Authors:** Gaofeng Liu, Nadeem Khan, Xiaoqing Ma, Xilin Hou

**Affiliations:** 1 State Key Laboratory of Crop Genetics & Germplasm Enhancement, Key Laboratory of Biology and Genetic Improvement of Horticultural Crops (East China), Ministry of Agriculture and Rural Affairs of the China, Engineering Research Center of Germplasm Enhancement and Utilization of Horticultural Crops, Ministry of Education of the China, Nanjing 210095, China; lgf@njau.edu.cn (G.L.); 2018204021@njau.edu.cn (X.M.); 2 Ottawa Research and Development Center, Agriculture and Agri-Food Canada, 960 Carling Avenue, Ottawa, ON K1A 0C6, Canada; 2016104235@njau.edu.cn; 3 Department of Biology, University of Ottawa, 30 Marie Curie, Ottawa, ON K1N 6N5, Canada

**Keywords:** Histone lysine methyltransferase, Histone demethylases, Co-regulatory analysis, Expression pattern, *Brassica rapa*

## Abstract

Histone modifications, such as methylation and demethylation, are vital for regulating chromatin structure, thus affecting its expression patterns. The objective of this study is to understand the phylogenetic relationships, genomic organization, diversification of motif modules, gene duplications, co-regulatory network analysis, and expression dynamics of histone lysine methyltransferases and histone demethylase in *Brassica rapa*. We identified 60 *SET* (HKMTases), 53 *JmjC*, and 4 *LSD* (HDMases) genes in *B. rapa*. The domain composition analysis subcategorized them into seven and nine subgroups, respectively. Duplication analysis for paralogous pairs of SET and JmjC (eight and nine pairs, respectively) exhibited variation. Interestingly, three pairs of SET exhibited *Ka/Ks* > 1.00 values, signifying positive selection, whereas the remaining underwent purifying selection with values less than 1.00. Furthermore, RT-PCR validation analysis and RNA-sequence data acquired on six different tissues (i.e., leaf, stem, callus, silique, flower, and root) revealed dynamic expression patterns. This comprehensive study on the abundance, classification, co-regulatory network analysis, gene duplication, and responses to heat and cold stress of SET and JmjC provides insights into the structure and diversification of these family members in *B. rapa*. This study will be helpful to reveal functions of these putative *SET* and *JmjC* genes in *B. rapa*.

## 1. Introduction

The role of histone methylation in transcription regulation was first reported in the 1960s [1]. It was not until 15 years later that a catalytic SET domain (Su(var)3–9, enhancer of zeste and trithorax) the first histone methyltransferase (*SUV39H1*), was identified [2]. This discovery led to a number of SET-domain homologous searches [3]. The subsequent discoveries of the key regulatory domain of demethylase enzymes [4], JmjC (Jumonji C), substantially expanded the repository of histone demethylases. Histone methylation and demethylation play fundamental roles in various biological processes, specifically in regulating transcription, genome integrity, and epigenetic inheritance [5,6]. Histone modification is a complex process occurring on various residues (lysine and arginine) and at different sites with the addition of a varying number of methyl groups. Mass spectroscopy and liquid chromatography were used to identify the histone modification profile in *Arabidopsis thaliana*, which revealed both conserved and non-conserved modifications compared to animals [7,8]. Active or repressive transcription is conferred liable on the positions and methylation states, unlike other histone modifications, in which active or repressed chromatin states are specified [9]. More often, methylation is considered as active transcription (such as in H3K4, H3K36, and H3K79), although some methylation processes are assumed to be connected with silenced chromatin states like H3K9, H3K27, and H4K20 [9]. Thus, post-transcriptional histone modifications play a pivotal role in activating and silencing genes of various important biological processes in eukaryotes [10].

Histone lysine methyltransferase (HKMTases) contains the SET domain while histone demethylase (HDMases) is comprised of two major domains namely JmjC and LSD (lysine-specific demethylase domains) [11]. There are seven classes in *A. thaliana* based on amino acid sequence features and domain compositions of SET [12,13]. These SET domain protein members play a pivotal role in chromatin regulation, structure, and function [14,15]. In *Arabidopsis* SU(VAR)3-9 members have H3K9 methyltransferase activity that plays a vital role in heterochromatin formation and gene silencing [16,17,18]. While SET proteins catalyze H3K27 trimethylation and are largely known in the repressive control of gene expression [19]. Similarly, JmjC proteins also play crucial roles in regulating epigenetic processes during growth and development [20] and many members of JmjC have been characterized in different species. The LSD domain, on the other hand, has small members, present only in four genes in *A. thaliana*.

Some of the recent studies highlighted the response of methylation in response to cold and heat stress. In *Arabidopsis* on the cold-responsive genes such as *COR15A*, and *AtGolS3*, decreased gradually in both a histone occupancy-dependent and independent manner during cold stress treatment [21]. In maize, histone acetylation in cold-responsive genes including *ZmDREB1* and *ZmCOR413* increased [22]. Similarly, in rice the *OsDREB1b* genes was induced by cold stress [23]. The global methylation in response to heat varies between species. The exposure of *Arabidopsis* to heat stress results in an increased global methylation and higher homologous recombination frequency [24]. In *Arabidopsis,* the upregulation of *DRM2*, nuclear RNA polymerase D 1(*NRPD1*) and *NRPE1* in response to heat stress may contribute to increased genome methylation [25]. An increase in global methylation is also observed in *Cork oak* (*Quercus suber* L.) grown in higher temperatures [26]. In *Brassica napus*, the DNA methylation levels increase more in the heat-sensitive genotype compared to heat-tolerant genotype under heat treatment [27]. Thus, epigenetic changes induced by cold stress and heat are likely to contribute to the cold stress tolerance through changes in the expression profiling of genes.

The research detailed above emphasizes the need to further explore the importance of histone methylation and its complex nature. In addition, the evolutionary implications and functional dissection of these family members in *B. rapa* will enhance understanding of their functions and regulatory mechanisms. Most angiosperms have undergone polyploidization during their long evolutionary history [28]. In parallel with the polyploidization, as a result of whole genome duplication (WGD) events in the angiosperm genome, the two large gene families (HKMTases and HDMases) experienced development and functional diversity. Moreover, maintenance of genes at high numbers as a network or as a dose-sensitive response proves the gene dosage hypothesis [29]. Chinese cabbage (*B. rapa*) is an excellent model plant for genome evolution study due to the availability of its sequenced and assembled genome [30]. *B. rapa* also underwent two duplication (WGD α and β) and one whole-genome triplication events (WGT γ) in the *Brassicaceae* lineage [31]. The WGT led to widespread fractionation in the *B. rapa* genome, thus providing an opportunity to understand the molecular evolution of *SET* and *JmjC* genes in *B. rapa*. Its extensive data is also closely related to *A. thaliana*, estimating that about 93% of the predicted gene families of *B. rapa* appeared in *A. thaliana* and making it a vital model for evolutionary and genomic studies [32].

To date, 198, 136, 124, and 71 genes coding for histone methylation proteins have been identified using bioinformatics surveys in apple, sweet orange, tomato, and strawberry, respectively [33,34,35,36]. Previously, 67 and 49 genes from *B. rapa* were identified as SET candidates [37,38], however, important features such as the role of abiotic stress, and tissue expression patterns and correlation analysis along with the evolutionary analysis of paralogous pairs were not explored. In this work, the genes of *HKMTases* and *HDMases* from various clades are denoted as BraSET, BraJmjC, and BraLSD. A systematic and comprehensive description of the histone lysine modifiers in *B. rapa* was carried out through a comparative genome analysis to investigate copy variation, gene retention, syntenic region, collinear correlation, and expansion patterns following the WGT event. Furthermore, phylogenetic relationships, promoter sequence analysis, divergent tissue-specific expression patterns across six various tissues, co-regulatory network analysis, and the response of these genes to heat and cold stress will allow further understanding of functional analysis of *SET* and *JmjC* genes in *B. rapa*.

## 2. Results

### 2.1. Identification of (SET, JmjC, LSD) and Their Classification Patterns in Brassica rapa

In this study, we identified 60 *BraSET*, 53 *BraJmjC*, and 4 *BraLSD* genes in *B. rapa*. To validate our results for domain confirmation, these proteins were submitted to the SMART and NCBI servers. These proteins were designated based on their respective family information and following the nomenclature of *A. thaliana* according to sequence similarities, i.e., *BraSET1–BraSET60*, *BraJmjC1–BraJmjC53*, and *BraLSD1–BraLSD4* (Table S1). In addition, the protein length of BraJmjC and BraLSD vary widely, ranging from 171 to 2591 and 731 to 1579 (aa), compared to BraSET, which vary from 152 to 2396 (aa) (Table S1). The molecular weight of the resulting proteins ranged from 17.76 to 273.33 kDa, 20.29 to 293.09, and 76.43 to 174.28, respectively, and the corresponding pIs were 4.78 to 9.72, 4.83 to 8.82, and 5.15 to 6.71. These results varied among family members. These proteins may function in different microenvironments. The grand average of hydropathicity (GRAVY) for these proteins was observed to be negative, i.e., they exhibited hydrophilic behaviour. Subcellular localization is important for understanding plant functions, and our findings depicted that large proportion of the proteins were located in the plasma membrane, cytoplasm, nucleus, vacuoles, and mitochondria.

### 2.2. Expansion and Structural Features of SET and JmjC

**Figure 1:**
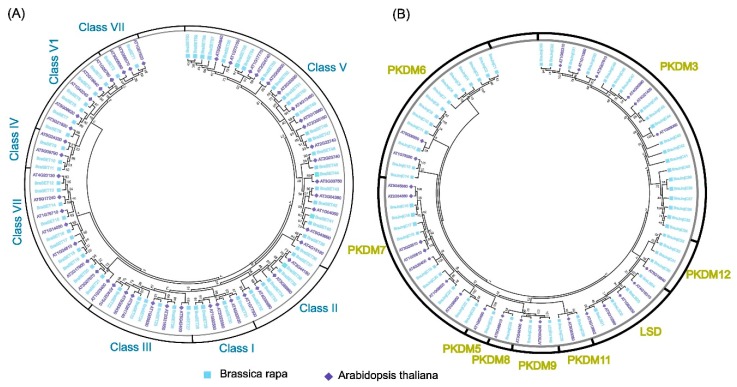
Phylogenetic relationship of SET (**A**) and JmjC (**B**) between *B. rapa* and *A. thaliana*. The phylogenetic tree was constructed by MEGA 7 using the Maximum Likelihood Method (1000 bootstrap). Genes of *B. rapa* and *A. thaliana* are marked with different colors.

A phylogenetic tree was constructed to compare and validate the evolutionary relationships of SET and JmjC proteins among *B. rapa* and *A. thaliana*. The maximum likelihood approach was used for both with 1000 bootstrap replications (Figure 1a). We assigned the SET proteins into seven subclasses, which was consistent with a previously reported study on *A. thaliana* [13]. Class V was the most dominant among all of the subclasses with 23 genes, while the other subclasses had relatively lower numbers of SET (Figure 1a). According to a previous characterization [39], *LSD* genes contain two domains, SWIRM and amino oxidase domains. Thus, the proteins containing both of these domains were categorized as putative LSD (JmjC), according to previously reported study [36]. Moreover, JmjCs were further subcategorized into different subgroups; LSD, PKDM06, PKDM07, PKDM08, PKDM09, PKDM11, PKDM12, and PKDM3, and PKDM5 based on phylogenic relationship and domain composition (Figure 1b). The result of our phylogenetic tree analysis was consistent with previous reports [40,41]. The highest number of genes (20) was observed in the subgroup PKDM3 of *B. rapa*, while the other groups contained lower numbers of JmjC (Figure 1b). Intriguingly, LSD contained four genes each in *B. rapa* and *A. thaliana*, suggesting that these genes may not have contributed to the duplication events during the process of evolution in the angiosperms. The execution of MEME program identified ten conserved motifs, named motifs 1–10 for both SET and JmjC (Figures S1a and S2a). The gene structure organization showed uniformity and consistency for both SET and JmjC (Figures S1b and S2b). We acquired the LOGO of these motifs for both SET and JmjC. Most of the subclasses of SET and JmjC had similarities in their motif arrangements. Both or one of motifs 1 and 2 were common among all the members, indicating they are a highly conserved domain. The consensus sequence showed greater extent of variability between SET and JmjC (Figures S3 and S4). In SET, motifs 3 and 5 revealed greater consensus sequence numbers (120), whereas remaining motifs varied in their consensus sequence number. We also observed divergence among subgroups for both SET and JmjC, which may be due to difference in tree topologies, leading to slight variations.

### 2.3. Gene Retention and Collinearity Analysis of SET and JmjC

**Figure 2:**
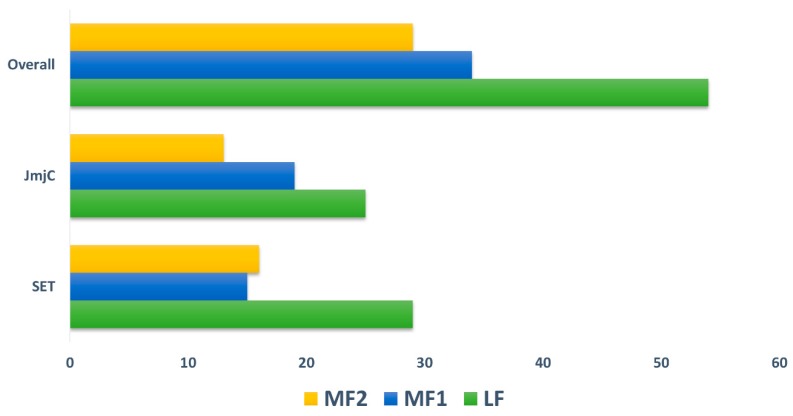
The ratio SET and JmjC, and their overlap among three subgenomes (i.e., LF = Least fractionated, MF1 = Medium Fractionated and MF2 = Most Fractionated genome) of *B. rapa*.

The *B. rapa* genome is classified into three subgenomes including least fractionated (LF), medium fractionated (MF1), and most fractionated (MF2) and shares a diploid ancestor with *A. thaliana* [31]. The gene retention and copy number of variations for both SET and JmjC were investigated and calculated in both *B. rapa* and *A. thaliana* during a *Brassica*-specific WGT event. We similarly analyzed the syntenic relationship between paralogous and orthologous gene pairs by utilizing the BRAD database (Table S2). The retention of both SET and JmjC depicted that many of the proteins are either in a single or double copy among three subgenomes. Moreover, one pair with four copies was found for SET and three pairs with three copies were identified for JmjC, whereas the rest did not show any copy variation (Table S2). Our results also indicated that the gene retention and both gene families presented almost identical results, 60/61 SET, and 57/58 JmjC. As discussed earlier, the *B. rapa* genome contains three subgenomes according to their fractionation degree for SET, the LF subgenome included most of the genes (48.33%), followed by MF2 (26.67%) and MF1 (25%). We noted slight variation for JmjC as the LF share was only 43.86%, followed by MF1 (33.33%) and MF2 (22.81%). Overall, the results confirmed that large proportion of the genes (46.15%) were sited in the LF subgenome of *B. rapa* (Figure 2). Our results validate the hypothesis of dosage prediction as high number of gene copies were retained, indicating that high degree of gene retention occurred following a WGD event.

### 2.4. Chromosomal Localization and Syntenic Gene Duplication Analysis

**Figure 3:**
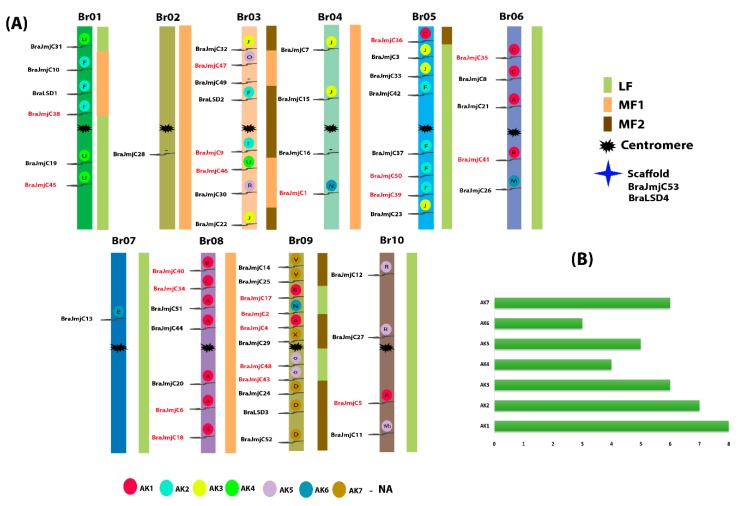
(**A**) Chromosome locations of JmjC were obtained from the GFF file and displayed using Mapchart. The paralogous pairs are displayed in red and while the three subgenomes (i.e., LF = Least fractionated, MF1 = Medium Fractionated and MF2 = Most Fractionated genome) of *Brassica rapa* are also visualized by different color. The ancestral karyotypes (i.e., AK1–AK7) are marked in different colors and the letters inside the circles represent the block positioned in the genome of *B. rapa*. (**B**) It represents the proportion of AK in *B. rapa* genome.

To explore the evolutionary mechanism of both SET and JmjC, the chromosomal location and gene duplication events were further analyzed. For chromosomal localization, all SET and JmjC displayed an obvious uneven dispersal on the 10 different positions of *B. rapa* chromosomes (Bra01–Br10) (Figure S5a and b and Figure 3a). A large number of SET were recorded on chromosome Br09 (up to 16) while the lowest number was found on chromosomes Br01 with two genes. For JmjC the maximum number of chromosomes was also observed on Bra09 (up to 11) and the minimum number was noted on Bra02 and Bra07 with a single each gene. Four genes were unmapped and were distributed on a scaffold—two of each gene form SET (*BraSET41* and *BraSET42*) and JmjC (*BraJmjC53* and *BraLSD4*). In addition, we reconstructed the 24 conserved ancestral genomic blocks (GBs) mainly based on a previously reported study [42]. In a proposed ancestral karyotype (AK), their position determined the color-coding of these blocks [43,44], with slight modification. Most of the SET were clustered in the regions of AK1 and AK3 (18.92% each), whereas for JmjC, most (20.51%) were clustered in the region of AK1 as shown in Figure 3b.

**Figure 4:**
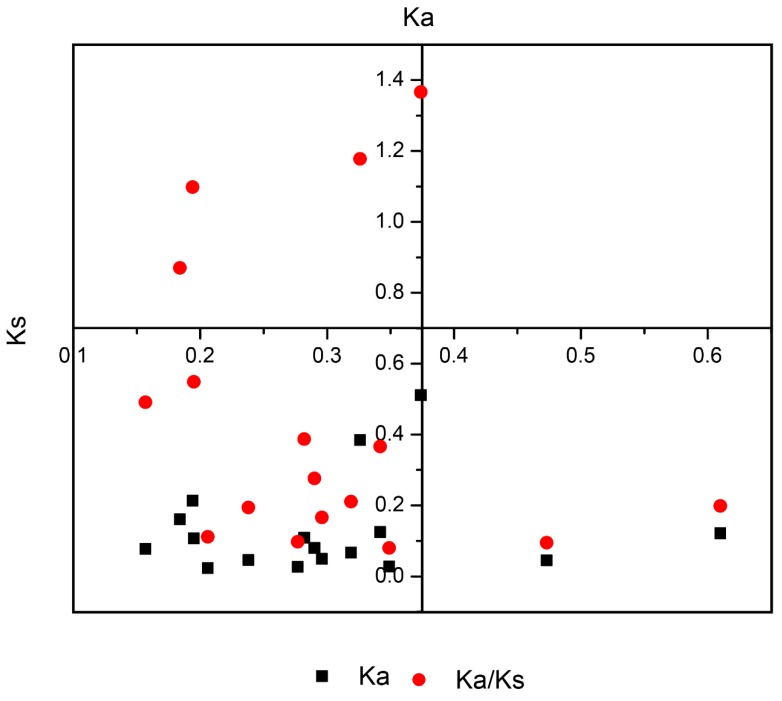
The ratio between *Ks* and *Ka* for paralogous gene pairs in *Brassica rapa*.

For a gene family’s evolution, gene duplication is the driving force for gene activation [45]. Using MCScanX software, we detected duplication types (tandem and segmental). In *B. rapa*, the expansion of SET and JmjC was mainly driven by WGD events or segmental duplication. In SET genes, the *Ks* values ranged from 0.16 to 0.61 with an average divergence time of 11.13 MYA (million years ago). For JmjC, the *Ks* values ranged from 0.18 to 0.35 with an average divergence of 9.04 MYA (Figure 4 and Table S3). Taken together, these values suggest that it mainly initiated with the divergence of *B. rapa* from *A. thaliana* (9.6–16.1 MYA) [46].

### 2.5. Expression Patterns of SET and JmjC in Various Tissues

**Figure 5:**
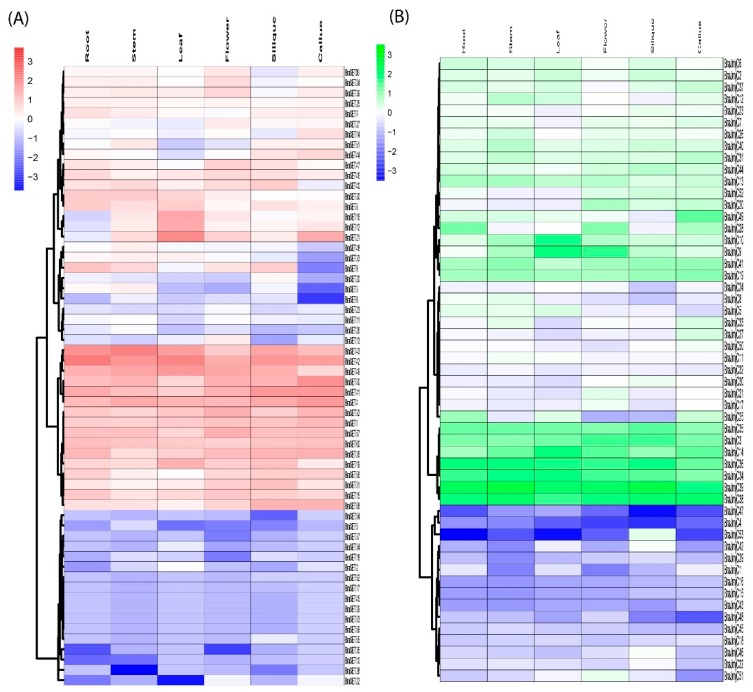
Heat map of expression profiles (log_2_ fold-change) for SET (**A**) and JmjC (**B**) in the six various tissues: stem, flower, callus, silique, root, and leaf. The expression levels are indicated by the color bar.

Previously published RNA-seq data on various tissues (i.e., roots, stems, leaves, flowers, siliques, and callus) was retrieved and used to gain insight into the divergence and putative functions of *SET* and *JmjC* genes in *B. rapa* growth and development [30]. Results demonstrated higher discrepancies in expression profiling of SET and JmjC gene members in *B. rapa*. Among 60 *SET* genes, *BraSET29*, *BraSET45*, and *BraSET53* are not expressed and *BraSET17*, *BraSET52*-*55*, and *BraSET59* showed random involvement in a particular organ (Figure 5A and Table S4). The remaining SETs were expressed in at least two or more organs. Few genes were selectively expressed in tissue-specific clustering (Figure S6A). Among them, one gene in flowers and five in siliques showed preferential expression patterns and could be selected as candidate genes for likely role in tissue improvement of *B. rapa.*

The data showed that 57 *JmjC* genes were expressed highly compared to SET; only one gene (*BraJmjC43*) did not show any expression pattern, while the rest were expressed highly in one or more tissues (Figure 5C and Table S4). In tissue-specific clustering (Figure S6B), we observed only two selective genes in siliques, suggesting their possible role in organ developmental pathways.

The expressional tendencies between 17 paralogous pairs were investigated for both SET and JmjC (eight and nine pairs, respectively), and their correlation values were calculated (Figure S7A and Table S5). Results revealed that paralogous pairs presented substantial distinction in six different tissues. Eight pairs of SET showed varied expression, including three pairs (*BraSET6_BraSET9, BraSET58_BraSET59, and BraSET57_BraSET60*), showing higher expression in all organs with positive correlation (>0.6). In addition, two pairs (*BraSET45_BraSET46* and *BraSET53_BraSET55*) showed no correlation and the rest were expressed with mild positive correlation. These results indicated that due to pseudogenization, these genes might have lost function. On the other hand, for JmjC, four pairs (*BraJmjC5_BraJmjC6*, *BraJmjC9_BraJmjC10*, *BraJmjC34_BraJmjC35*, and *BraJmjC40_BraJmjC41*) showed higher expression in all tissues with higher PCC values (>0.6), one pair (*BraJmjC43_BraJmjC48*) had no correlation, one (*BraJmjC17_BraJmjC18*) exhibited negative correlation, and the rest showed mild positive correlation. In addition, the clustering image showed that only one gene participated in siliques, while the rest were not co-expressed in other tissues (Figure S7B). The divergences in the expression profiles for both SET and JmjC between paralogous pairs suggest that few of these pairs may gain new functions following the duplication during evolutionary process.

### 2.6. Cis-Element and Expression Analysis of SET and JmjC

The promoter regions of SET and JmjC was used for the identification of cis-regulatory elements by using the PlantCARE database. A total of seven major groups, such as light, hormones, stress factors, enhancers, other regulatory stress factors, and circadian were the most prominent promoter region and found to be conserved among SET and JmjC domains. For both SET and JmjC, we identified the number of genes that were responsible for various cis-regulatory elements (Table S6). These results demonstrated that numerous genes were involved in various signaling pathways, for instance, certain genes (39.62%) were light-responsive (L-BOX, ATI-motif, ATC-motif, AE-BOX, MRE, G-BOX, GAG-motif, and LAMP-ELEMENTS), followed by hormones (16.44%) (CGTCA, ABRE, ERE-motif, TGACG-motif, P-BOX, TGA, AuxRR-core, GARE-motif), and other regulatory stress elements (15.66%) (ARE, AT-Rich sequence, A-Box, GCN4-motif, CAT-BOX, o2-site, EIRE), while few genes (3.29% and 2.51%) participated in enhancers (GC-motif, 5UTR Py-rich stretch, and TA-Rich Region) and circadian. These results suggest that both SET and JmjC were highly responsive to light stress factors, which may be due to interaction with corresponding cis-elements that assist in regulating gene expression levels. There were some other common regulatory elements such as heat stress-responsive (HSE), drought responsible (MBS), low temperature responsible (LTR), and drought, cold, and salt stress-responsive elements (DRE), which indicate the diversity in function and the importance of these key genes in stress tolerance mechanisms.

**Figure 6:**
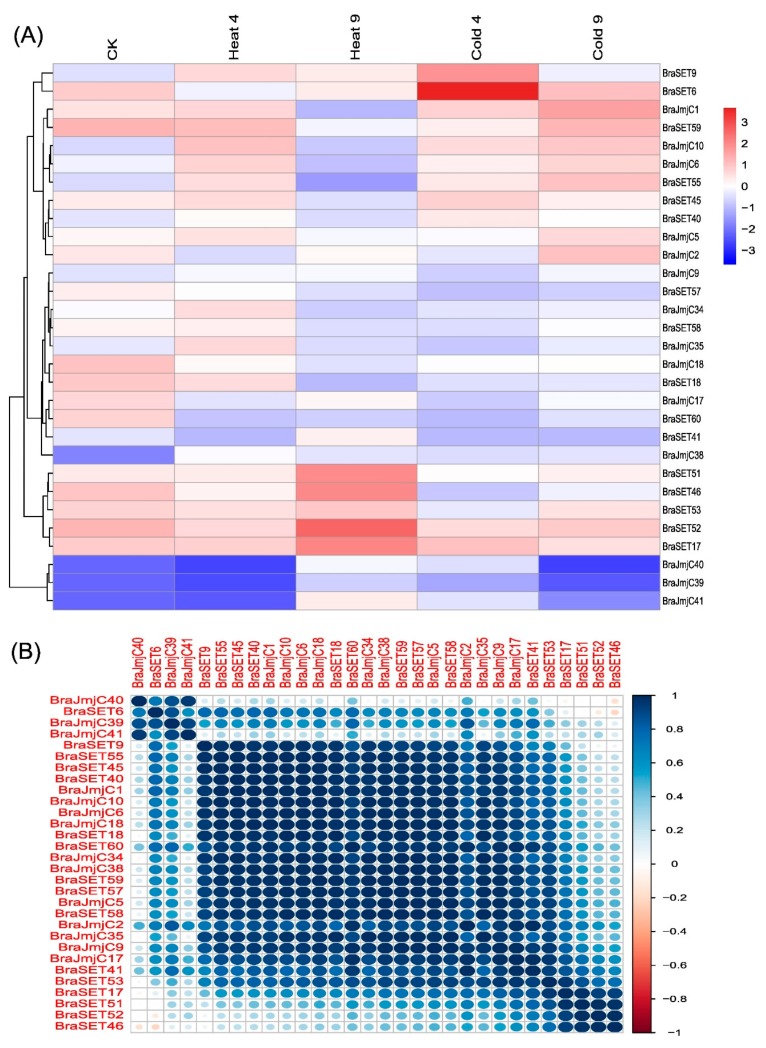
(**A**) Relative expression (log_2_ fold-change) analysis of *SET* and *JmjC* genes under CK, heat and cold stress treatments in *B. rapa*. (**B**) Pearson’s correlation coefficients (PCCs) of relative gene expression of 15 paralogous pairs used for RT-PCR analysis during heat and cold stress treatments.

In angiosperms and vertebrates, histone modifications, such as methylation and demethylation, have been identified as crucial factors for regulating chromatin structure. As a result, both the *JmjC* and *SET* genes play a crucial role in developmental stages to tolerate abiotic stresses [41,47]. The importance of these genes and the results of cis-regulatory elements provide the means to study these genes’ dynamic expression under heat and cold stress. We randomly selected a total of 15 paralogous pairs of genes, including eight SET and seven JmjC, and analyzed them using qRT-PCR. A range of differential expression levels were observed after exposure to heat and cold stress (Figure 6A). Some of the paralogous pairs for both SET and JmjC exhibited similar patterns, whereas others showed significant variations. Under the two abiotic treatments (heat and cold), more than half of genes were induced by heat and exhibited striking expression patterns compared to the cold treatment. The PCCs based correlation analysis suggested both high positive and low negative correlation within selected genes (Figure 6B).

## 3. Discussion

To understand the gene structure, function, and evolution, a genome-wide gene family analysis is the first step [47]. Moreover, for the identification of histone modifiers in a genome, sequence-based searching and phylogenetic characterization are the most effective methods [41,48,49]. We performed a comprehensive search for SET and JmjC domain-containing genes throughout the *B. rapa* genome, and a total of 60 and 57 full-length *SET* and *JmjC* genes were identified, respectively. These genes were further divided into seven SET and nine JmjC distinct subgroups based on domain organization and phylogenetic analysis. The phylogenetic characterization of SET and JmjC was highly conserved among subgroups, suggesting the importance of their roles in regulatory mechanisms for plant improvement. SET (Class V) and JmjC (PKDM3) groups were preferentially expanded in *B. rapa* compared to *A. thaliana,* implying that these group members evolved substantially to meet some unique regulatory needs [36]. The result of our study is consistent with similar work on *A*. *thaliana*, *O. sativa* and *Citrus* [34,50]. This comprehensive analysis on phylogenetic relationships, syntenic regions, and the collinear relationship between *B. rapa* and *A. thaliana* also demonstrated that *BraSET, BraJmjC*, and *BraLSD* were divergent from the model plant at a high frequency.

Evolutionary history indicated that multiple polyploidization events have occurred in all extant angiosperms [51,52,53]. During the evolutionary process and genetic systems, gene duplications are the main driving force for novel biological functions, extensive development of the gene family, and the generation of evolutionary novelty [54]. *B. rapa* is tremendous model plant to study evolutionary process as it has undergone WGD and WGT events, which allows the study of the relationships among gene family fractionation and discrepancies in morphotypes [31,42]. As discussed earlier, angiosperms experienced polyploidization events in the evolutionary process, which led to the expansion of *SET* and *JmjC* genes in *B. rapa* resulting from the WGT event to allow non-functionalization, sub-functionalization, and neo-functionalization [55]. For duplicated types of genes, these fates help them by providing options to gain functional diversification. The analysis of duplication types revealed more segmental duplication (91.08%) compared to tandem, indicating that segmental duplication plays a significant role in the contribution and expansion of SET-domain, JmjC-domain, and LSD-domain proteins. Our findings also demonstrated a high occurrence of copy variation and gene retention following a WGT event, which supported the gene dosage hypothesis [29]. Furthermore, we also calculated the divergence rate for paralogous SET and JmjC. The comparison of nucleotide distance showed that JmjC diverged at approximately 9.04 MYA, earlier than SET which separated at 11.13 MYA. The divergence time for both SET and JmjC for paralogous gene pairs indicates that their divergence occurred during the divergence of *B. rapa* from *A. thaliana* (9.6–16.1 MYA) [45]. Generally, if the value of *Ka/Ks* is < 1, indicates gene pairs may have evolved from purifying selection (also called negative selection); *Ka/Ks* = 1 suggests neutral selection, while *Ka/Ks* > 1 means positive selection [56,57,58]. Three pairs of SET had a *Ka/Ks* > 1, specifying positive selection, the remaining SET showed values less than 1.00. These results suggest that these pairs underwent purifying selection and thus mainly act the maintenance of *B. rapa*.

Comparative structural analysis of both SET and JmjC shared common patterns among subgroups. It is worth mentioning that gene structure compositions might be useful for studying the origin of these genes. We observed in most of the subclasses contained more than one intron, and the rest exhibited consistency among the subgroups, indicating that the distribution pattern of introns and exons is pondered as the backbone of genes and the evolutionary fingerprint [31]. Synonymous and non-synonymous analysis showed that the *SET* and *JmjC* genes did not differ significantly among the three subgenomes (LF, MF1, and MF2) of *B. rapa*. Our study offers insight into the unique features and fairly high conservation in *B. rapa.* To comprehend plant functions, expression analysis of a gene can provide valuable clues. Some of the emerging evidence has supports that cellular processes and epigenetic regulations commonly occur during abiotic stress, emphasizing the significance of both DNA and histone modifications [59,60]. In plants, the alteration of histone modification and DNA methylation are coordinated with changes in the expression profiling and that are associated with stress-responsive genes to adapt to environmental changes. Recent studies have reported that several histone modifications including H3K4me3, H3K9ac, H3K9me2, H3K23ac, H3K27ac, H3K27me3, and H4ac, along with DNA methylation response to abiotic stresses, such as drought stress, salt, and temperature fluctuations [61,62]. The response of plants to temperature stresses are categorized into different types based on the exposure such as warm, high, chilling or freezing temperature. The low-temperature stress and high-temperature stress greatly affects plant growth and development, immunity and circadian rhythm, and poses a major threat to the global food supply [63]. In *Arabidopsis*, the understanding of SUMOylation has been progressed under heat stress conditions and also various chromatin components including H2B, GCN5, HDA19, and the deubiquitinating enzyme UBP26, which removes ubiquinone bound to H2B, have been found to be SUMOylated [64]. The heat stress treatment has been reported to decrease the SUMOylation of H2B and increase the status of the GCN5 HAT [64]. In *Arabidopsis*, vernalization processes involved in epigenetic regulation induced by environmental stresses and can be achieved by long-term exposure to cold temperatures [65]. In *Arabidopsis*, it has been estimated that 3 to 20% of the transcription changes occurred in response to cold stress [66,67]. The expression of HDA6 in *Arabidopsis* was induced by long-term low temperature treatments that resulted into mutation by showing sensitivity to freezing stress [68]. Expression profiling for 15 SET and JmjC paralogous pairs was analyzed after exposure to heat and cold treatments by qRT-PCR. The results, specifically for heat and cold stress genes, showed a high variation in the expression profile and provided valuable clues for robust candidate genes in improving stress tolerance mechanisms in *B. rapa.* The paralogous gene pairs respond differently. For most *SET* genes, the expression level increases after heat shock and 9 pairs showed a positive correlation. By contrast, the JmjC responded mostly negatively to cold treatment. In summary, some *SET* genes show active expression patterns upon heat and cold shock, which specifies that these genes may be participated in *B. rapa* responses to temperature stresses. The overall response of SET and JmjC across six various tissues was revealed by the combinatorial expression profiles against heat and cold stress. Certainly, the different organs/stages have dynamic expression patterns in specific tissues or treatments, and many of those *SET* and *JmjC* genes showed variation either up- or downregulation in definite tissues or treatments. Cis-elements and expression analysis results revealed that *SET and JmjC* genes expressed in various biotic, abiotic and hormone signaling, might have acquired new functions after duplication in the evolutionary processes. A common interacting pattern was observed in the expression profiling such as cis-acting element involved in heat stress-responsiveness (HSE) and in low-temperature responsiveness (LTR) as described in Table S6. Both *SET* and *JmjC* genes showed variable responses to these factors and can be assumed to be responsive against heat and cold stress.

Cumulatively, our results provide fundamental information about SET-domain, JmjC-domain, and LSD-domain protein members that will assist in the identification and for functional studies in boosting abiotic stress-resistant crop plants.

## 4. Material and Methods

### 4.1. Retrieval of Data Sequences

The sequences were downloaded from *B. rapa* genome (2.5 version), BRAD (http://brassicadb.org/brad/) [31]. The *A. thaliana* sequences were retrieved from TAIR (http://www.arabidopsis.org/) and the sequences of rice were extracted from the Rice Genome Annotation Project (http://rice.plantbiology.msu.edu/) [69]. The Hidden Markov Model profile (HMM) was used as a query in our study to find homologous proteins based on the domain information. For various families, such as SET (PF00856), JmjC (PF02373), SWIRM (PF04433), and Amino_oxidase (PF05193), the HMM file was downloaded from the Pfam 31.0 database, (https://pfam.sanger.ac.uk/) [70]. These potential protein sequences were manually analysed with the help of SMART (http://smart.embl-heidelberg.de/) [71] and NCBI databases (https://www.ncbi.nlm.nih.gov/). Proteins that contained both SWIRM and Amino_oxidase domains were identified as *LSD* genes. Sequences with obvious errors including gene length or domain compositions were eliminated.

### 4.2. Multiple Sequence Alignment (MSA) and Phylogenetic Analysis

For MSA of SET and JmjC candidate genes, we executed MUSCLE [72] by MEGA 7 software with the default options [73]. The phylogenetic trees were constructed using the maximum likelihood (ML) method. For the reliability of resulting tree, bootstrap values of 1000 replications were performed with the Jones, Taylor, and Thornton amino acid substitution model (JTT model), while keeping the other parameters as a default.

### 4.3. Calculation of the Ka/Ks ratio

The *Ka/Ks* ratios for the paralogs of SET and JmjC were calculated using MEGA 7.0 [73] software and was intended using the Nei-Gojobori method (Juke-Cantor) with 1000 bootstrap replicates. Additionally, the paralogous genes were identified by searching the term ‘syntenic region’ in the *B. rapa* genome. Gene pairs were selected among the subgenomes (i.e., LF = Least fractionated genome, and MF1 = Medium fractionated genome or MF2 = Most fractionated genome) of *B. rapa*. The rate of divergence was calculated by using the following formula: T = *Ks*/2r, where *Ks* represents the synonymous substitutions per site and r is the rate of divergence. For dicotyledonous plants, specifically *B*. *rapa*, the hypothesis is 1.5 synonymous substitutions per site of 10^8^ years [74].

### 4.4. Conserved Motifs, Exon–Intron Structure Analysis, and Physicochemical Parameters of SET and JmjC Proteins

To identify the conserved motifs for both SET and JmjC proteins, Multiple Em for Motif Elicitation (MEME) software version 5.0.5 was used with the following parameters: maximum number of motifs 10, with a minimum width of 100 and a maximum of 120. The other parameters were set as default [75]. We used the Gene Structure Display Server (GSDS 2.0) (http://gsds.cbi.pku.edu.cn) for exon–intron structure [76]. The physicochemical properties of the proteins, such as molecular weight (MW), isoelectronic points (pI), and grand average of hydropathicity (GRAVY) values for each gene, were calculated using the PROTPARAM tool (http://web.expasy.org/protparam/). Finally, WOLF PSORT (https://wolfpsort.hgc.jp/) server was used to predict subcellular localization.

### 4.5. Cis-Elements and Proteins Interaction Predictions

The promoter sequences of SET and JmjC (selected as 2000 upstream bp) were retrieved from the *B*. *rapa* genome according to the generic file format (GFF). The various cis-elements were recognized by using the PlantCARE database (http://bioinformatics.psb.ugent.be/webtools/plantcare/html/) [77].

### 4.6. Chromosomal Location and Synteny Analysis of SET and JmjC

The chromosomal location of SET and JmjC was illustrated from top to bottom with respect to their position in the genome annotation using Mapchart [78]. For gene synteny analysis, the relationships were verified between the homologs of *A. thaliana* and subgenomes of *B. rapa* (LF, MF1, and MF2) obtained from BRAD (http://brassicadb.org/brad/searchSynteny.php). Circos program was applied to demonstrate the syntenic relationships among the chromosomes of *B. rapa* and *A. thaliana* [79]. Moreover, the paralogous pairs were recognized by either selecting gene pairs between LF and MF1 or MF1 and MF2 subgenomes of *B. rapa*.

### 4.7. Pearson Correlation Coefficient (PCC)

Pearson correlation coefficient for both RNA-seq and qRT-PCR was performed by MS Excel (Ver. 2013) and figure was prepared using RStudio [80].

### 4.8. Plant Material and Treatments

The seeds of Chinese cabbage (Chiifu-401-42) were grown in plastic pots containing a mixture of soil and vermiculite (3:1) placed in a growth chamber for five weeks. The following growth conditions were maintained: the temperature was set to 24/16 ºC, the photoperiod was 16/8 h, and the relative humidity was 65–70%. Definite treatments (heat and cold) were provided to the seedlings as follows: for heat and cold treatments, seedlings were exposed to 38 ºC and 4 ºC, respectively and samples were collected with an interval of untreated (CK), 4 and 9 h, respectively. Finally, the samples were divided into three biological replicates and kept frozen in liquid nitrogen, and immediately stored at −70 ºC for further analysis.

### 4.9. RNA Extraction and qRT-PCR validation

For RNA extraction, frozen leaves from both treatments and control plants were sampled. RNA was isolated by using Trizol (Invitrogen, Carlsbad, CA, USA) method, following the manufacturer’s protocols. cDNA was prepared from both treatment and control groups by reverse-transcribing the RNA through Primer Script RT reagent kit (TAKARA, Dalian, China). The list and sequence of the primers used in our study are provided in Table S7. For the validation of the specificity of the primers, BLAST tool was used against the *B. rapa* genome. The RT-PCR assays were performed with three biological and three technical replicates by following the guidelines explained in our previous study with slight modifications [81]. In brief, each reaction was performed in a 20-μL reaction mixture containing a diluted cDNA sample as the template, 2 × Power SYBR Green PCR Master Mix (Applied Biosystems), and 400 nM each of forward and reverse primers. The reactions were performed using a MyiQ Single-Color Real-Time PCR Detection System (Bio-Rad, Hercules, CA, USA) with the following cycling profile: 94 °C for 30 s, followed by 40 cycles at 94 °C for 10 s, and 58 °C for 30 s. A melting curve (61 cycles at 65 °C for 10 s) was generated to verify the specificity of the amplification. For qRT-PCR analysis, we randomly selected 15 pairs of syntenic paralogs and the *B. rapa* actin gene *Bra028615* (Forward: CTCAGTCCAAAAGAGGTATTCT and Reverse: GTAGAATGTGTGATGCCAGATC) was used as an internal control for normalization. The relative fold expression was calibrated by using the comparative Ct-method. The gene expression levels for SET and JmjC were analyzed by following the previously reported study [30,82].

## 5. Conclusions

This study is an extensive genome-wide survey of the SET-domain, JmjC-domain, and LSD-domain proteins in *B. rapa*. We identified 60 *SET* and 57 *JmjC* genes by an in silico analysis of the *B. rapa* genome database. Phylogenetic analysis mapped closest putative orthologs of SET and JmjC from *B. rapa* and *A. thaliana* by sequence similarity, which were further divided into seven and nine subgroups, respectively. This classification was further supported by gene structure and motif analyses, with each group sharing a common junction of exon–intron and protein motifs. Transcriptomic analysis of *B. rapa* SET-domain, JmjC-domain, and LSD-domain proteins indicated that some of the family members exhibited tissue-specific expression. The expression analysis of *SET* and *JmjC* genes in response to heat and cold treatments indicated the co-occurrence of different signaling pathways. Our findings on genome-wide identification and expression analysis provide a foundation for the functional dissection of *SET*, *JmjC*, and *LSD*-domain in *B. rapa.*

## Supplementary Materials

The following are available online at https://www.mdpi.com/2223-7747/8/12/526/s1, Figure S1: Motif structure and gene structure for SET. (A) The CDS regions are represented by green boxes, respectively. (B) The conserved motifs of SET were determined by MEME with complete protein sequences. The color configuration specifies different motif numbers (1–10). At the bottom of the figure, the relative position is proportionally displayed based on the kilobase scale, Figure S2: Motif structure and gene structure for JmjC. (A) The CDS regions are represented by green boxes, respectively. (B) The conserved motifs of SET were determined by MEME with complete protein sequences. The color configuration specifies different motif numbers (1–10). At the bottom of the figure, the relative position is proportionally displayed based on the kilobase scale. Figure S3: The consensus sequence of conserved motifs of SET and predicted length (amino acids) for each motif. Figure S4: The consensus sequence of conserved motifs of JmjC and predicted length (amino acids) for each motif. Figure S5: Chromosome locations of SET were obtained from the GFF file and displayed using Mapchart. The paralogous pairs are displayed in red. The paralogous pairs are displayed in red and while the three subgenomes (i.e., LF = Least fractionated, MF1 = Medium fractionated and MF2 = Most fractionated genome) of *Brassica rapa* are also visualized by different color. The ancestral karyotypes (i.e., AK1–AK7) are marked in different colors and the letters inside the circles represent the block positioned in the genome of *Brassica rapa*. Figure S6: Venn diagram analysis of the tissue-expression of SET (A) and JmjC (B), Figure S7. (A) Heat map of expression profiles (log_2_ fold-change) for 17 SET and JmjC paralogous pairs in six various tissues: silique, stem, root, flower, leaf, and callus. The Pearson correlation coefficients (PCCs) are also displayed in brackets, while NA indicates no available results for PCC. (B) Venn diagram analysis of the tissue-expression of paralogous pairs for both SET and JmjC. Table S1: The basic description of *SET* and *JmjC* genes in *Brassica rapa*, Table S2: Identification of SET and JmjC syntenic genes between *A. thaliana* along with three subgenomes of *Brassica rapa*, Table S3: *Ka/Ks* calculation of the paralog pairs of SET and JmjC in *Brassica rapa*, Table S4: The FPKM values of *SET* and *JmjC* genes in *Brassica rapa*, Table S5: Syntenic paralog pairs of SET and JmjC with PC and FPKM values, Table S6: Cis-elements of *SET* and *JmjC* genes in *Brassica rapa*, Table S7: Sequences of *SET* and *JmjC* gene primers used for quantitative real-time PCR.
